# Supplementary material for: Genome assembly and functional predation analysis of novel Bdellovibrio isolates from human gut microbiota
Source: Front Microbiol. 2026 Feb 2;17:1752098. doi: 10.3389/fmicb.2026.1752098 (PMC12907353; doi:10.3389/fmicb.2026.1752098)
Supplement: Supplementary file 1 [file Table_1.docx]

# SUPPLEMENTARY MATERIAL of

**Cultivation and genomic characterization of human gut-associated *Bdellovibrio* reveals natural predatory bacteria with specialized prey interactions**

Mario Romero-Rivera^1,2,3*^, Miguel Díez-Fernández de Bobadilla^4*^, María Beltrán^5^, Rosa del Campo^1,6,7^✉, José Avendaño^1,6^✉, Cristina Herencias^1,6^✉

## **Supplementary Table S1.** Bacterial strains used in this study, their genotypes, antimicrobial resistance profiles, LPS modifications, and database accessions.

| **Strain** | **Genotype** | **AMR (phenotype / resistance genes)** | **LPS modification** | **Accession (NCBI / RefSeq / GenBank)** | **Reference** |
| --- | --- | --- | --- | --- | --- |
| ***B. bacteriovorus* HD100** | Wild type | None reported (non-pathogenic) | Native LPS; unmodified lipid A | NC_005363.1 | ATCC 15356 |
| ***P. putida* KT2440** | Wild type | Intrinsic efflux pump *mexAB-oprM* | Smooth LPS; no lipid A modification | NC_002947.4 | ATCC 47054 |
| ***P. aeruginosa* PAO ATCC 47085** | Wild type | Chromosomal *ampC*, *mexAB-oprM*, *oprD* | Canonical hexa-acylated lipid A | NC_002516.2 | ATCC  47085 |
| ***P. aeruginosa* PAOM** | *lpxL2* frameshift (ΔG328 → Glu110fs) | Similar intrinsic resistance as PAO1 | Altered lipid A acylation due to *lpxL2* mutation | -------- | Laboratory collection |
| ***E. coli* ATCC 25922** | Wild type | Fully susceptible; no acquired AMR genes | Smooth LPS; unmodified lipid A | CP009072.1 | ATCC 25922 |
| ***P. aeruginosa* 7** | Wild type (clinical isolate) | Colistin^S^, murepavadin^S^ | Addition of 4-amino-arabinose (Ara4N) to lipid A | -------- | (Avendaño-Ortiz et al., 2023) |
| ***P. aeruginosa* 7M** | *acrB2*, *cbrA* SNPs (T2726→C; A1919→G) | Colistin^R^  Murepavadin^R^ | Addition of 4-amino-arabinose (Ara4N) to lipid A | -------- | (Avendaño-Ortiz et al., 2023) |
| ***P. aeruginosa* 14** | Wild type (clinical isolate) | Colistin^S^  murepavadin^S^ | None detected | -------- | (Avendaño-Ortiz et al., 2023) |
| ***P. aeruginosa* 14M** | *lpxL1* deletion (C34 Ala12 fs) | Murepavadin^R^ | Altered lipid A acylation (defective lauroyl transferase) | -------- | (Avendaño-Ortiz et al., 2023) |
| ***P. aeruginosa* 28** | Wild type (clinical isolate) | Colistin^S^, murepavadin^S^ | None detected | -------- | (Avendaño-Ortiz et al., 2023) |
| ***P. aeruginosa* 28M** | Not reported | Murepavadin^R^ | Not detected (n.d.) | -------- | (Avendaño-Ortiz et al., 2023) |
| ***P. aeruginosa* 40** | Wild type (clinical isolate) | Colistin^S^ murepavadin^S^ | None detected | -------- | (Avendaño-Ortiz et al., 2023) |
| ***P. aeruginosa* 40M** | *lpxL2* deletion (C525→C559 Thr176 fs) | Murepavadin^R^ | Altered lipid A acylation due to *lpxL2* loss | -------- | (Avendaño-Ortiz et al., 2023) |
| ***K. pneumoniae* KPCS** | Wild type (colistin-susceptible isogenic pair) | Colistin-susceptible; no *mcr* genes detected | Wild-type lipid A (hexa-acylated) | -------- | (Díez-Aguilar et al., 2021) |
| ***K. pneumoniae* KPCR** | Mutations in *pmrAB* / *lpxM* | Colistin-resistant | Addition of 4-amino-arabinose (Ara4N) to lipid A phosphate groups | -------- | (Díez-Aguilar et al., 2021) |

S: susceptible; R: Resistant

## **Supplementary Table S2.** SNPs of predatory bacteria isolates BD_H1 and BD_H2 compared with the reference genome of *B. bacteriovorus* HD100

| **CHROMOSOME** | **POSITION** | **HD100** | **BD_H1** | **BD_H2** |
| --- | --- | --- | --- | --- |
| BX842601.2 | 1408 | C | G | G |
| BX842601.2 | 56910 | G | C | C |
| BX842601.2 | 57086 | T | C | C |
| BX842601.2 | 57191 | C | T | T |
| BX842601.2 | 219860 | C | T | T |
| BX842601.2 | 501128 | T | C | C |
| BX842601.2 | 501206 | T | C | C |
| BX842601.2 | 501219 | C | A | A |
| BX842601.2 | 501236 | T | C | C |
| BX842601.2 | 501251 | C | A | A |
| BX842601.2 | 501297 | T | C | C |
| BX842601.2 | 548330 | A | G | G |
| BX842601.2 | 889929 | C | G | G |
| BX842601.2 | 1298932 | C | T | C |
| BX842601.2 | 2119264 | A | T | T |
| BX842601.2 | 2119300 | C | T | T |
| BX842601.2 | 2119331 | G | A | A |
| BX842601.2 | 2138646 | G | C | C |
| BX842601.2 | 2138647 | T | G | G |
| BX842601.2 | 2138648 | G | T | T |
| BX842601.2 | 2353215 | T | T | C |
| BX842601.2 | 2886580 | A | G | G |
| BX842601.2 | 3579872 | C | T | T |
| BX842601.2 | 3606936 | T | C | C |
| BX842601.2 | 3606937 | T | G | G |
| BX842601.2 | 3735010 | G | A | A |

## **Supplementary Table S3.** Annotation of ribosomal RNA (rDNA) and transfer RNA (tRNA) genes identified in contigs 1 and 3 from predatory isolate from Bd_H2. All annotations are in the negative strand.

| **Sequence Id** | **Type** | **Start** | **Stop** | **Locus Tag** | **Gene** | **Product** | **DbXrefs** |
| --- | --- | --- | --- | --- | --- | --- | --- |
| contig_1 | rDNA | 58 | 174 | LOEBGI_00005 | rrf | 5S ribosomal RNA | GO:0003735, GO:0005840, KEGG:K01985, RFAM:RF00001, SO:0000652 |
| contig_1 | rDNA | 430 | 3370 | LOEBGI_00010 | rrl | 23S ribosomal RNA | GO:0003735, GO:0005840, KEGG:K01980, RFAM:RF02541, SO:0001001 |
| contig_1 | tRNA | 3484 | 3560 | LOEBGI_00015 | trnI | tRNA-Ile(gat) | SO:0000263 |
| contig_1 | rDNA | 3703 | 5216 | LOEBGI_00020 | rrs | 16S ribosomal RNA | GO:0003735, GO:0005840, KEGG:K01977, RFAM:RF00177, SO:0001000 |
| contig_3 | rDNA | 21 | 135 | BGAEOE_00005 | rrf | 5S ribosomal RNA | GO:0003735, GO:0005840, KEGG:K01985, RFAM:RF00001, SO:0000652 |
| contig_3 | rDNA | 263 | 3142 | BGAEOE_00010 | rrl | 23S ribosomal RNA | GO:0003735, GO:0005840, KEGG:K01980, RFAM:RF02541, SO:0001001 |
| contig_3 | tRNA | 3367 | 3443 | BGAEOE_00015 | trnI | tRNA-Ile(gat) | SO:0000263 |
| contig_3 | tRNA | 3474 | 3549 | BGAEOE_00020 | trnA | tRNA-Ala(tgc) | SO:0000254 |
| contig_3 | rDNA | 3642 | 5187 | BGAEOE_00025 | rrs | 16S ribosomal RNA | GO:0003735, GO:0005840, KEGG:K01977, RFAM:RF00177, SO:0001000 |

## **Supplementary Table S4. 163 *Bdellovibrio* genome identifiers from NCBI**

## **Supplementary Table S5.** Genome similarity between the 17 *Bdellovibrio* strains with ANI > 94%.

| **Strain** | **Target** | **Similarity** | **organism_name** |
| --- | --- | --- | --- |
| HD100 | GCA_000196175.1_ASM19617v1 | 0.9910326 | *B. bacteriovorus* |
| 109J | GCA_000691605.1_ASM69160v1 | 1 | *B. bacteriovorus* |
| 109J | GCA_037076595.1_ASM3707659v1 | 1 | *B. bacteriovorus* |
| -- | GCA_040377645.1_ASM4037764v1 | 0.9797863 | *B. bacteriovorus* |
| HD100_Sm | GCA_040746155.1_Bba-HD100-Sm | 0.9910326 | *B. bacteriovorus* |
| MHI39 | GCA_040746165.1_ASM4074616v1 | 0.9910326 | *B. bacteriovorus* |
| MHI353 | GCA_040746175.1_ASM4074617v1 | 0.9910326 | *B. bacteriovorus* |
| MHI182 | GCA_040746185.1_ASM4074618v1 | 0.9910326 | *B. bacteriovorus* |
| MHI179 | GCA_040746195.1_ASM4074619v1 | 0.9910326 | *B. bacteriovorus* |
| MHI167 | GCA_040746265.1_ASM4074626v1 | 0.9910326 | *B. bacteriovorus* |
| MHI154 | GCA_040746335.1_ASM4074633v1 | 0.9910326 | *B. bacteriovorus* |
| MHI153 | GCA_040746365.1_ASM4074636v1 | 0.9910326 | *B. bacteriovorus* |
| Bd13 | GCA_041888765.1_ASM4188876v1 | 0.9907545 | *B. bacteriovorus* |
| YOA24 | GCA_048286975.1_ASM4828697v1 | 0.9503546 | *B. bacteriovorus* |
| LR-3 | GCA_050312355.1_ASM5031235v1 | 0.9893455 | *B. bacteriovorus* |
| BD_H1 | consensus_cov_Pool1.fna | 0.9910326 | *B. bacteriovorus* |
| BD_H2 | consensus_cov_Pool2.fna | 0.9908742 | *B. bacteriovorus* |

## **Supplementary Table S6.** Predatory activity is expressed as plaque-forming units (PFU) in each depredation replicate assay done for BD_H1, BD_H2, and *B. bacteriovorus* HD100 against a panel of laboratory reference strains and clinical isolates. Each predator-prey combination was tested in three to five independent replicate predation assays .

## **Supplementary Figures**

**
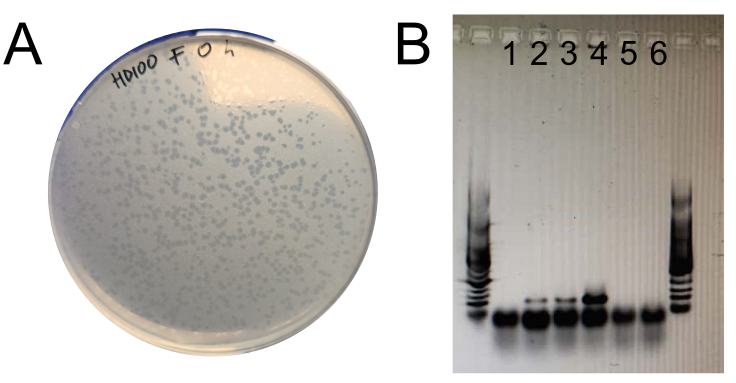
**

**Supplementary Figure S1.** A) After incubation of 72-96 h of incubation on double-layer agar culture containing *P. putida*, *B. bacteriovorus* HD100 formed circular plaques. B) PCR amplification targeting the Bdellovibrionaceae family was performed using specific primers Bd347F and Bd549R using the plaques of the plates corresponding to pools 1 to 5. (1: negative control, 2: *B. bacteriovorus* HD100 as positive control, 3: plate formed from pool1, 4: plate formed from pool2, 5: plate formed from pool4, 6: plate formed from pool5).


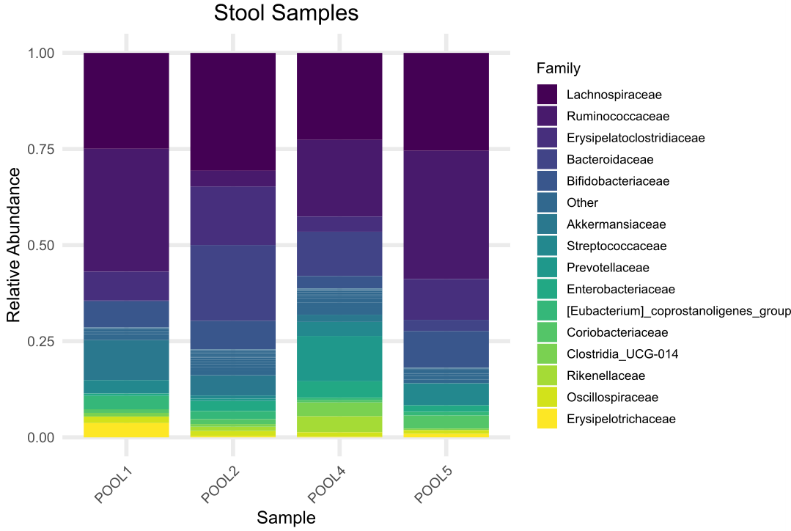


**Supplementary Figure S2.** Bacterial community composition at the family level of the five pooled human fecal samples before enrichment, determined by 16S rDNA gene amplicon sequencing. Despite high sequencing depth, no Bdellovibrionaceae sequences were detected above the threshold (>10 reads), indicating their extremely low natural abundance in the human gut microbiota.


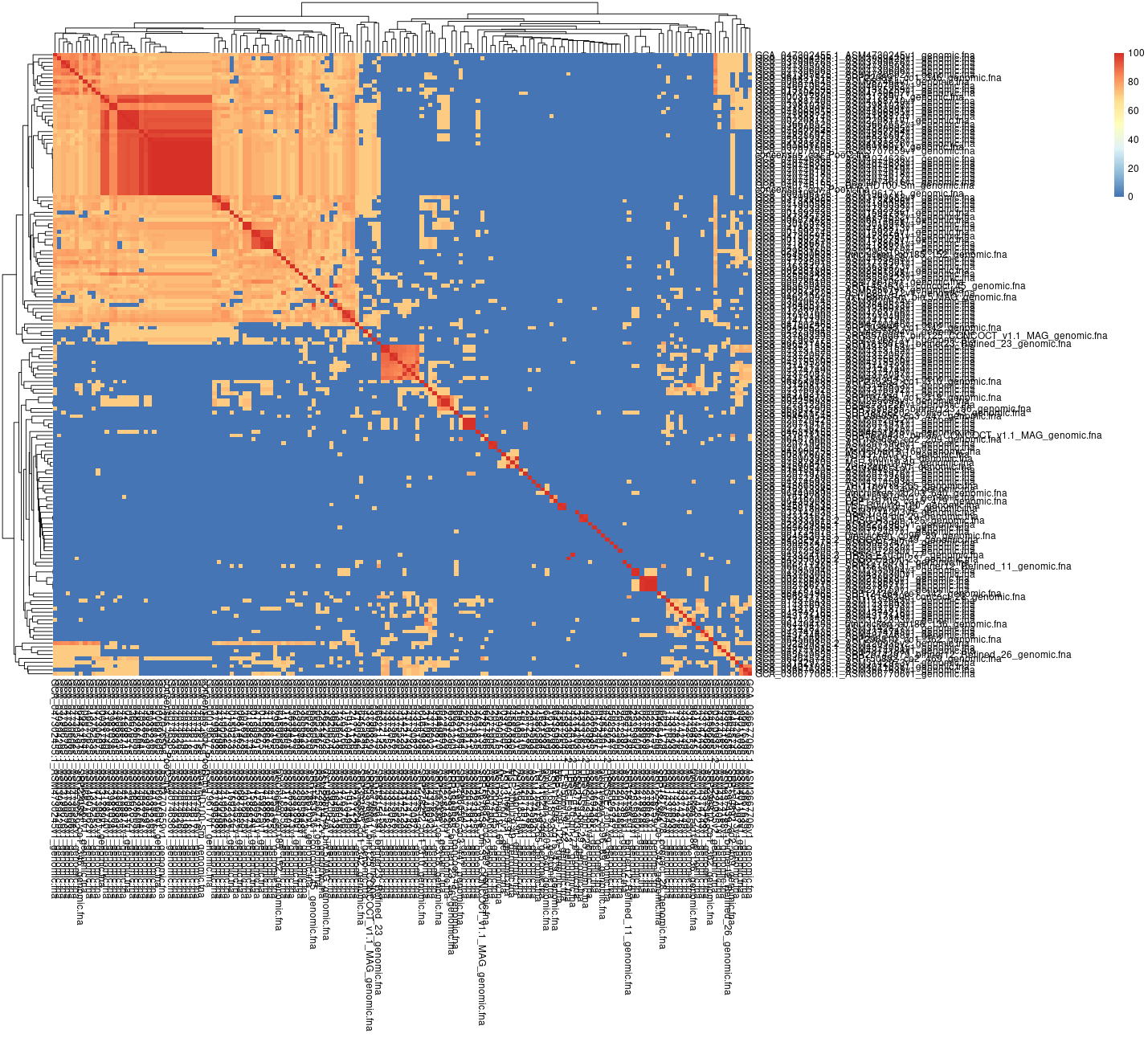


**Supplementary Figure S3. Genome-wide average nucleotide identity (ANI) heatmap of 163 *Bdellovibrio* spp. genomes retrieved from NCBI.** Pairwise ANI values were calculated using the PATO pipeline and are displayed as a clustered heatmap with hierarchical clustering based on genomic similarity. The color scale represents ANI percentage (blue = 0%, red/orange = 100%). Human-derived isolates (consensus_cov_Pool1.fna= BD_H1 and consensus_cov_Pool2.fna=BD_H2) are indicated. Genome accession numbers are identified in Supplementary Table 4.


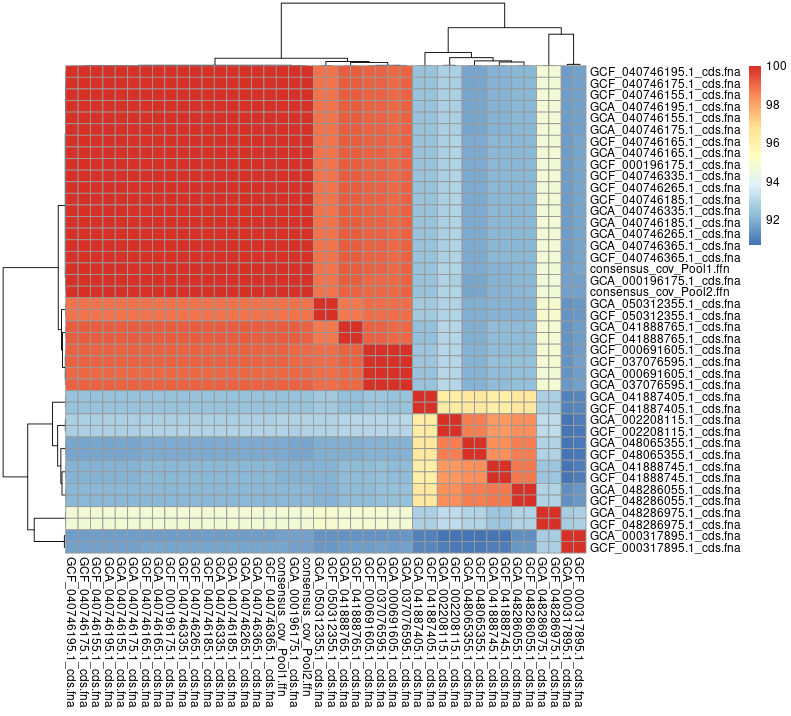


**Supplementary Figure S4.** Pairwise ANI heatmap of *Bdellovibrio* and closely related predatory bacteria (≥90% ANI; n=41). Human-derived isolates (consensus_cov_Pool1.fna= BD_H1 and consensus_cov_Pool2.fna=BD_H2) are indicated. Genome accession numbers are identified in Supplementary Table 4.


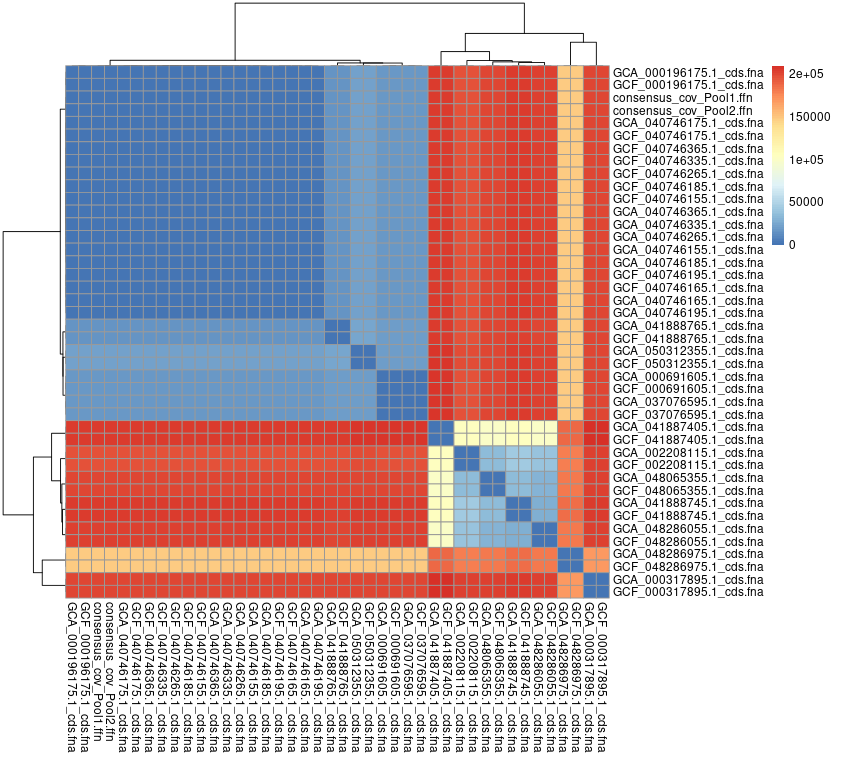


**Supplementary Figure S5. Core genome single nucleotide polymorphism (SNP) distribution across *Bdellovibrio* species.** Hierarchical clustered heatmap showing pairwise SNP counts from recombination-filtered core genome alignments across 41 *Bdellovibrio* genomes (≥90% ANI; n=41). Blue indicates genomic conservation; red indicates SNP divergence. Human-derived isolates (consensus_cov_Pool1.fna= BD_H1 and consensus_cov_Pool2.fna=BD_H2) are indicated. Genome accession numbers are identified in Supplementary Table 4.
